# Predictive value of preoperative platelet count and D-dimer levels for spinal cord injury following acute type a aortic dissection

**DOI:** 10.1186/s13019-024-02597-y

**Published:** 2024-03-13

**Authors:** Fengbo Pei, Jinhua Wei, Yao Yao, Hui Wu, Zujun Chen

**Affiliations:** 1 Cardiovascular surgery department, Fuwai Hospital, Chinese Academy of medicai sciences and Peking Union Medical College, National Center for Cardiovescular Diseases, Beijing, China; 2 Peking University People’s Hospital, Cardiac surgery department, Beijing, China

**Keywords:** Spinal cord injury, Acute type a aortic dissection, Platelet, D-dimer

## Abstract

**Background:**

This study aims to identify the risk factors contributing to spinal cord injury (SCI) following a type A acute aortic dissection (TA-AAD).

**Methods:**

This retrospective study was conducted at a single center and involved 481 patients who received frozen elephant trunk stent implantation for TA-AAD. Additionally, these patients underwent total arch replacement with deep hypothermic circulatory arrest. This study was performed at Fuwai Hospital between September 2016 and April 2020.

**Results:**

The resulting data of the multivariate logistic regression analysis demonstrated that preoperative platelet count (odds ratio [OR] = 0.774) and D-dimer levels (OR = 2.247) could serve as independent predictors for postoperative SCI in patients with TA-AAD.

**Conclusion:**

The findings indicate that preoperative platelet count and D-dimer levels are independent risk factors for postoperative SCI in patients with TA-AAD. This study holds significant clinical implications regarding the prognosis and therapeutic responses for patients with TA-AAD.

## Background

Type A acute aortic dissection (TA-AAD), a clinically fatal and acute medical condition, exhibits a pre-hospital mortality rate of approximately 49%. This condition is acknowledged as one of the most pressing surgical emergencies in cardiovascular surgeries [1–4]. The pathological impact of this dissection may extend to multiple organs, including the spinal cord, leading to ischemia and consequential postoperative spinal cord injury (SCI) as notable complications. These complications, with an incidence ranging from 3.5 to 20% [5], can adversely affect the prognosis of patients and their quality of life following surgery. Recent advancements, particularly the extensive application of the total arch replacement (TAR) coupled with the frozen elephant trunk (FET) technique, have led to a gradual decline in the mortality rate associated with TA-AAD. Nonetheless, SCI remains a severe postoperative complication. Therefore, it may be potentially beneficial to reduce the incidence of SCI through early identification of patients at high risk for postoperative SCI during the preoperative period, thereby enabling more proactive and intensive interventions in their treatment regimen.

## Methods

### Patient enrollment

This study involved a cohort of patients with Stanford TA-AAD admitted to the Fuwai Hospital, Chinese Academy of Medical Sciences and Peking Union Medical College (Beijing, China) from September 2016 to April 2020. A retrospective analysis was conducted using the electronic medical records and laboratory test results of these patients. However, exclusion criteria for the study were applied to patients who did not receive TAR, such as isolated ascending aorta replacement or partial aortic arch replacement, those who did not undergo deep hypothermic circulatory arrest (DHCA) techniques like hybrid total aorta replacement, or those with chronic type A aortic arch dissection.

### Study design

This single-center retrospective study was designed to analyze the patients with TA-AAD who underwent TAR with DHCA using the FET technique. The primary focus of this study was to explore the preoperative characteristics, intraoperative details, and postoperative outcomes of these patients. This study was approved by the Ethics Committee of the Fuwai Hospital, Chinese Academy of Medical Sciences and Peking Union Medical College (Beijing, China). Informed written consent was obtained from each participant or their caregivers. The diagnosis of TA-AAD in this study was determined through enhanced computed tomography (CT) scanning. In addition, this research examined the incidence of postoperative SCI in patients with TA-AAD and identified the risk factors associated with this complication.

### Surgical procedures

Conventional anesthetic techniques, including endotracheal intubation, were employed throughout the surgical procedure. Cardiopulmonary bypass (CPB) was applied *via* cannulation of the right axillary artery. Intraoperatively, DHCA was applied, along with selective implementation of either unilateral or bilateral cerebral perfusion, based on clinical requirements. TAR was performed using four-branch prosthetic grafts involving the re-anastomosis of the right innominate brachiocephalic trunk, left common carotid artery, and left subclavian artery to the corresponding branches of the prosthetic vessels. Furthermore, the FET stent was surgically implanted into the descending aorta. The stents used for the FET procedure during surgery were acquired from Endovastec (Shanghai, China). The appropriate length of the stent was selected based on the presence or absence of a distal tear in the descending aorta, ensuring that the FET adequately covered any distal tear in the descending aorta. Standard sizes of 28 mm × 100 mm and 28 mm × 120 mm were routinely used. The cerebrospinal fluid drainage (CSF) strategy at our center involved routine CSF drainage for all patients with type A aortic dissection affecting the descending aorta. In the emergency room, CSF drainage via lumbar puncture was immediately performed for patients at high risk for paraplegia due to dissection. For patients with type A aortic dissection not at high risk for paraplegia, CSF drainage was routinely conducted after anesthesia in the operating room.

SCI was classified according to the criteria established by the American Spinal Injury Association (ASIA; https://asia-spinalinjury.org/) into five grades. Grade A represented a complete injury, while Grade B denoted an incomplete injury characterized by sensory function below the neurological level of injury, albeit without any motor function. Grade C indicated an incomplete injury where more than half of the motor functions were preserved below the neurological level of injury, yet with crucial muscle strength not exceeding Level 3. Grade D also represented an incomplete injury, but with more than half of the motor function maintained below the neurological level of injury. Grade E was assigned if both motor and sensory functions were normal. In the ASIA classification, Grade A reflected the most severe SCI, namely paraplegia, while the spinal cord at Grade E was considered normal.

### Statistical analysis

The distribution of data was assessed using the Kolmogorov-Smirnov test. Normally distributed continuous data were presented as mean ± standard deviation (SD), whereas non-normally distributed continuous data were summarized using the median value and interquartile range. Categorical variables were expressed as counts and percentages and analyzed using the Chi-square or Fisher exact tests. One-way analysis of variance or Wilcoxon rank sum test was utilized for the comparison of continuous variables. The logistic regression model was used to identify the univariate and multivariate predictors of postoperative SCI. All analyses were performed using SPSS 26 (SPSS, IBM Corp. Armonk, USA), with *p* less than 0.05 representing statistical significance.

## Results

### Demographic characteristics

A total of 774 patients with TA-AAD were enrolled through the inpatient electronic information system. Among them, 293 were excluded due to their surgical procedures not aligning with the inclusion criteria of the study. This exclusion included 226 cases of partial aortic arch replacements without DHCA, 45 cases of hybrid total aortic arch procedures, and 22 cases of chronic type A aortic arch dissections. Finally, 481 cases of TA-AAD were selected for the study, with a mean age of 47.2 ± 16.4 years (29–75 years). This cohort consisted of 425 male and 56 female patients. Among the patients, 22 had aortic dissection tears originating from the aortic sinus, 174 had tears beginning in the ascending aorta, 247 had dissection tears originating from the aortic arch, and 38 patients had dissection tears that started in the descending aorta and extended retrogradely to the aortic arch.

Notably, 39 patients developed SCI postoperatively, with an incidence rate of 8.1%. Among these, 21 patients were diagnosed with paraplegia, while the remaining 18 had incomplete SCI. The preoperative demographic characteristics of both groups are shown in Table 1. Regarding demographic data and preoperative indicators, the two groups demonstrated no significant statistical differences in such variables as age, body mass index (BMI), time from onset to surgery, preoperative complications, preoperative ultrasound results, levels of preoperative leukocytes and hemoglobin, and the number of involved intercostal vessels. However, notable differences were observed in gender distribution, preoperative platelet counts, preoperative D-dimer levels, and the involvement of the Adamkiewicz artery.

**Table 1:** Preoperative demographic characteristics of patients with TA-AAD

Demographic characteristics	Normal group (*n* = 442)	SCI group (*n* = 39)	P
Demographic information			
Age	46.9 ± 17.2	50.7 ± 17.9	0.084
Gender (male)	396 (89.5%)	29 (74.3%)	0.009
BMI	24.9 ± 3.2	26.7 ± 4.1	0.612
Onset to surgery time (h)	45[21,124]	42[19,140]	0.194
Medical history			
Hypertension	407 (92.0%)	36 (92.3%)	0.795
Diabetes	11 (2.4%)	2 (5.1%)	0.645
Cerebrovascular disease	21 (4.7%)	3 (7.6%)	0.670
Smoking history	224 (50.6%)	23 (58.9%)	0.408
COPD	2 (0.4%)	0	> 0.999
Preoperative situation			
Lactic acid (mmol/L)	1.9 ± 0.7	2.1 ± 0.9	0.091
PaO_2_/FiO_2_	265 ± 82	249 ± 76	0.163
D-dimer (µg/mL)	1.1[0.4,2.9]	2.7[0.8,3.4]	0.004
Leukocytes (10^9/L)	12.7 ± 2.4	11.6 ± 3.5	0.071
Hemoglobin (g/L)	134 ± 21	131 ± 25	0.647
Platelets (10^9/L)	179 ± 28	144 ± 21	< 0.001
Blood creatinine (µmol/L)	87.5 ± 29.4	90.6 ± 30.4	0.117
Troponin I	0.02[0.00,0.06]	0.02[0.00,0.08]	0.911
Aortic root diameter (mm)	42.1 ± 8.4	42.9 ± 10.2	0.379
Ascending aorta diameter (mm)	45.8 ± 7.6	46.7 ± 8.1	0.317
Aortic arch (mm)	39.7 ± 6.5	40.1 ± 7.3	0.254
Proximal descending aorta (mm)	29.7 ± 4.8	31.4 ± 5.7	0.089
Left ventricular ejection fraction (%)	62.5 ± 6.4	63.8 ± 5.8	0.453
Adamkiewicz artery involvement	89 (20.1%)	16 (41.0%)	0.004
Intercostal arteries originating from the false lumen	299 (67.6%)	22 (70.9%)	0.153
Number of involved intercostal vessels	3 [0,6]	4 [0,7]	0.847

Note: TA-AAD, type A acute aortic dissection; SCI, spinal cord injury; BMI, body mass index; COPD, chronic obstructive pulmonary disease;

The intraoperative conditions of the two groups are presented in Table 2. No statistical difference was noted between the two groups concerning surgery duration, intraoperative CPB duration, aortic cross-clamp time, antegrade selective cerebral perfusion time, visceral ischemia time, and intraoperative nasopharyngeal temperature. However, intraoperative blood loss and CSF pressure were substantially higher in the SCI group compared to the control group.

**Table 2:** Intraoperative characteristics of patients with TA-AAD

Features	Normal group (*n* = 442)	SCI group (*n* = 39)	P
Surgical procedures			
Bentall + TAR-FET	51 (11.5%)	7 (17.9%)	0.120
CABG + TAR-FET	29 (6.5%)	4 (10.2%)	0.466
Bentall + CABG + TAR-FET	6 (1.3%)	1 (2.5%)	0.477
Surgery duration (h)	8.7 ± 1.9	9.2 ± 2.2	0.068
CPB duration (min)	174 ± 52	180 ± 57	0.059
Aortic cross-clamp time (min)	139 ± 46	144 ± 57	0.228
Antegrade selective cerebral perfusion time (min)	32 ± 7	30 ± 5	0.410
Visceral ischemia time (min)	21 ± 4.7	22 ± 5.4	0.314
Nasopharyngeal temperature (℃)	24.7 ± 2.9	24.2 ± 4.5	0.773
Intraoperative blood pressure (MAP, mmHg)	51 ± 7.3	50 ± 6.9	0.220
Intraoperative blood loss (mL)	1071 ± 446	1453 ± 579	0.035
Intraoperative CSF pressure (mmHg)	14.7 ± 5.4	19.5 ± 7.6	0.015

Note: TA-AAD, type A acute aortic dissection; SCI, spinal cord injury; CPB, cardiopulmonary bypass; DHCA, deep hypothermic circulatory arrest; CSF, cerebrospinal fluid

Postoperative conditions of both groups are shown in Table 3, indicating a slightly higher in-hospital mortality rate in the SCI group than the normal group (12.8% vs. 2.9%). Furthermore, postoperative complications, including prolonged stays in the intensive care unit (ICU), the necessity for renal replacement therapy, occurrences of low cardiac output, and gastrointestinal complications, were notably more prevalent in the SCI group relative to the normal group.

**Table 3:** Postoperative outcomes of patients with TA-AAD

Outcomes	Normal group (*n* = 442)	Paraplegic group (*n* = 39)	P
In-hospital death within 30 days	13 (2.9%)	5 (12.8%)	0.007
ICU stay (days)	5 [3,11]	9 [6.17]	0.001
Postoperative renal replacement therapy	26 (5.8%)	11 (28.2%)	< 0.001
Re-thoracotomy	4 (0.9%)	0	0.746
Postoperative low cardiac output	5 (1.1%)	6 (15.3%)	< 0.001
Postoperative gastrointestinal complications	14 (3.1%)	5 (12.8%)	0.011
PND (stroke and cerebral hemorrhage)	21(4.7%)	5(12.8%)	0.032
AF	82(18.6%)	6(15.3%)	0.577
Pneumonia	22(4.9%)	4(10.2%)	0.162
Sepsis	21(4.7%)	2(5.1%)	0.915
Wound infection	5(1.1%)	1(2.5%)	0.439
MOF	19(4.2%)	3(7.6%)	0.330
IABP	0	0	
ECMO	5(1.1%)	1(2.5%)	0.454

Note: ICU, intensive care unit; AF, atrial fibrillation; MOF, multiorgan failure; IABP, intra-aortic balloon pump; ECMO, extracorporeal membrane oxygenation; PND, permanent neurological dysfunction

In the univariate logistic regression analysis, preoperative, intraoperative, and postoperative factors were evaluated. Subsequently, multivariate logistic regression analysis was conducted on variables with a *P* value < 0.1. Based on the backward-stepwise regression method, factors with a *P* value ≥ 0.1 were removed from the regression model in each step. After adjusting for confounders, the results are shown in Table 4. The analysis identified preoperative platelet count (odds ratio [OR] = 0.774, 95% confidence interval [CI] 0.416–0.895, *P* = 0.007) and D-dimer levels (OR = 2.247, 95% CI 1.756–4.226, *P* = 0.016) as independent risk factors for SCI after TA-AAD. Other variables that remained significant in the multivariate logistic regression model included gender (OR = 3.117, *P* = 0.004), intraoperative CSF pressure (OR = 1.816, *P* = 0.029), and postoperative low cardiac output (OR = 2.149, *P* = 0.017).

**Table 4:** Risk factors in multivariate logistic regression analysis of postoperative paraplegia in patients with TA-AAD

Variable	OR	95% CI	P
Gender	3.117	1.592–8.063	0.004
Preoperative platelet count	0.774	0.416–0.895	0.007
Preoperative D-dimer levels	2.247	1.756–4.226	0.016
Intraoperative CSF pressure	1.816	1.469–3.274	0.029
Postoperative low cardiac output	2.149	1.736–5.075	0.017

Note: TA-AAD, type A acute aortic dissection; OR, odds ratio; CI, confidence interval; CSF, cerebrospinal fluid

## Discussion

The present study revealed that a decline in preoperative platelet count and an elevation in D-dimer levels were significant independent risk factors for postoperative SCI. Despite the advancements in surgical techniques, the treatment of TA-AAD remains a complex and challenging endeavor in the surgical field. Nevertheless, with the increasing adoption of TAR using the FET technique, there is a noted decrease in both mortality and the necessity for re-thoracotomy in these cases. SCI and paraplegia, as severe complications, profoundly impact the prognosis of patients, not only increasing their mortality but also adversely affecting long-term quality of life. The reported incidence of SCI in TA-AAD patients, as stated in the literature, shows a wide range from 3.5 to 20% [5].

Prior research has primarily focused on intraoperative surgical procedures and intraoperative spinal cord protection strategies, such as the reconstruction of the intercostal vessels and Adamkiewicz artery. Some studies suggest that intraoperative circulatory arrest, accompanied b a core body temperature ≥ 28 °C, a duration > 40 min, and insufficient cerebral protection, may contribute as risk factors for postoperative SCI. Whether the FET technique leads to insufficient blood supply to the spinal cord, thereby elevating the risk of SCI, continues to be a topic of discussion. Chakos et al. [7] have reported an increased incidence of SCI in procedures using the FET technique compared to conventional aortic arch repair (CAR) using a soft elephant trunk (FET: 3.8% vs. CAR: 1.4%, *P* < 0.01). Contrary to these findings, the study conducted by Ling et al. [8] did not corroborate this observation. According to their observations, the FET stent did not elevate the risk of postoperative paraplegia, and the “cutoff” sign after descending aortic surgery might be a critical factor leading to early postoperative paraplegia. Several studies have proposed that excessive coverage of the intercostal artery by the distal anchorage of the FET stent, especially below the Th10 level, may pose a risk factor for SCI [9–10]. However, this hypothesis has not been consistently supported by other studies [11]. According to the study of Hori et al. [6], higher creatinine levels and significant perfusion of the spinal cord through the false lumen were identified as potential contributors to the risk of postoperative SCI.

The spinal circulation is a longitudinal, continuous, and adaptable system [12, 13], implying that the contribution from any single segmental artery along its course is not critically vital. The spinal cord circulation is susceptible to disturbances in the initial days following extensive embolization of segmental arteries. Disruptions in the equilibrium of spinal cord blood supply due to hypovolemia, hyperthermia, and high venous pressure should be avoided. Once the anterior spinal artery proliferates and new vessels are generated and converted into large arteries, the spinal cord blood supply is expected to return to normal. Therefore, rapid thrombosis of the preoperative false lumen leading to reduced spinal arterial blood supply may be a pivotal risk factor for postoperative SCI.

Notably, this study identified that preoperative platelet count decline and preoperative D-dimer level elevation were risk factors for postoperative SCI. The mechanism might involve the massive platelet depletion attributed to preoperative thrombosis within the false lumen of the dissection. The persistent decline in platelets often suggests the exacerbation of aortic dissection in patients. A study by Liu et al. [14] has suggested an association between a decline in postoperative platelet count with aortic dissection and increased 3-year mortality.

Rapid thrombosis within the false lumen of aortic dissection can result in reduced blood flow through the intercostal arteries, with an inability for timely compensatory collateral circulation, potentially serving as a crucial factor in spinal cord ischemia. Prior research has demonstrated that in the acute phase of aortic dissection, thrombosis in the false lumen can lead to consumptive coagulopathy [15]. The pathogenesis of aortic dissection involves platelet activation, adhesion, and thrombus formation, leading to substantial platelet consumption. A decrease in platelet count reflects this significant depletion, potentially accompanied by the consumption of fibrinogen and other coagulation factors. An elevation in D-dimer levels also indicates accelerated platelet consumption. Preoperative platelet count may more accurately reflect the degree of thrombosis in the false lumen during the acute phase of aortic dissection. Clinically, we have consistently focused on patients with increased preoperative D-dimer levels and significant decreases in platelet count who are at high risk for paraplegia. Postoperatively, these patients are administered heparin anticoagulation to slow the progression of thrombosis in the false lumen and to promote the establishment of collateral spinal cord circulation. The efficacy of these methods in reducing the incidence of postoperative SCI requires further evaluation.

The D-dimer level is an indicator of coagulation in the body. It is recognized as a by-product of cross-linked fibrin degradation in humans. It assumes a significant role in the anticoagulation system of the body, and this role is crucial for maintaining the permeability of the vascular walls and normal blood flow. Furthermore, D-dimer is pivotal in the processes of coagulation and fibrinolysis and is commonly used as a serum biomarker to reflect these processes in patients. As previously reported, D-dimer reflects the fibrinolytic process of coagulation, and its level is linked to the size of the thrombus and the contact area with blood [16]. Wang et al. [17] also demonstrated that D-dimer levels were considerably elevated in patients with type A aortic dissection after massive thrombosis of the false lumen than in type B dissection. Additionally, it was observed that patients who succumbed to this condition exhibited notably higher D-dimer levels than those who survived.

### Limitations

This study has certain limitations. The data were retrieved from a single medical center with a relatively small size for this retrospective study, which may have introduced selection bias. Additionally, this study did not consider factors such as the individual experience of surgeons and variations in treatment philosophies of different medical institutions, which might have influenced the results. Hence, further prospective studies are still needed to substantiate these results.

## Conclusion

The findings of this study suggest that a reduction in preoperative platelet count and an elevation in D-dimer level are two independent risk factors for postoperative SCI in patients with TA-AAD undergoing TAR using the FET technique. Therefore, proactive management strategies incorporating suitable preventive measures are recommended for these patients to mitigate the risk of postoperative SCI.

## Data Availability

The data generated during and/or analyzed during the current study are available from the corresponding author upon reasonable request.
